# Contrasting Behavioural and Biochemical Characteristics of Normal and Spontaneously α‐Synuclein‐Deficient Mice Treated With MPTP

**DOI:** 10.1111/jnc.70201

**Published:** 2025-08-18

**Authors:** W. H. Powell, A. Ghita, F. C. Pascut, K. F. Webb, A. Newman‐Tancredi, M. M. Iravani

**Affiliations:** ^1^ Department of Clinical, Biological and Pharmaceutical Sciences University of Hertfordshire Hatfield UK; ^2^ Optics & Photonics Research Group, Faculty of Engineering University of Nottingham Nottingham UK; ^3^ Neurolixis SAS Castres France

**Keywords:** 1‐methyl‐4‐phenyl‐1,2,3,6‐tetrahydropyridine (MPTP), α‐synuclein, α‐synuclein deficiency, astrocytes, dopamine neuron, GFAP, IBA‐1, microglia, Parkinson's disease model, Raman spectroscopy

## Abstract

α‐Synuclein is the primary toxic constituent of Lewy bodies, but its exact function under homeostatic conditions remains elusive. To better understand the role of α‐synuclein, we compared two C57BL sub‐strains: the normal α‐synuclein‐expressing J6 and the α‐synuclein‐deficient J6‐OlaHSD, for behavioural, dopaminergic and glial integrity in substantia nigra (SN) and caudate putamen (CPu) before and after 1‐methyl‐4‐phenyl‐1,2,3,6‐tetrahydropyridine (MPTP) treatment. After MPTP treatment, J6 mice showed significant weight loss (−7% by Day 10), whereas OlaHSD mice maintained stable body weight. At baseline, J6 mice exhibited 33% higher locomotor activity but 38% more thigmotaxis, and 33% less endurance on the Rotarod test than OlaHSD mice. Loss of tyrosine hydroxylase‐positive neurons was similar in OlaHSD (−40%) and J6 mice (−34%). J6 mice had double the SN GFAP‐ir cells of J6‐OlaHSD, a difference that was unchanged by MPTP treatment. In the CPu, MPTP increased GFAP‐ir cells in both strains, but Iba1‐ir cells significantly increased only in MPTP‐treated OlaHSD mice, compared to J6 strain. We further compared the biochemical signatures using Raman micro‐spectroscopy. The Raman spectra of the freshly cut SN sections showed a greater shift in the α‐helix to β‐sheet protein conformation ratio in MPTP‐induced J6 mice, likely due to the absence of the *Snca1* gene in OlaHSD mice. These findings suggest that the absence of α‐synuclein plays a subtle role in the behavioural and neurochemical differences but has no significant effect on dopaminergic neurotransmission. It is therefore concluded that the presence of α‐synuclein is important for non‐dopaminergic behaviours such as anxiety‐like behaviours and regulation of body weight. Under toxic challenge, gliosis in the SN and CPu may be regulated by α‐synuclein. This study also emphasises the utility of Raman spectroscopy as a potential tool for identifying subtle protein conformation differences in mice with and without *Snca1*.

## Graphical abstract

α‐Synuclein's role under normal conditions is unclear. We compared α‐synuclein‐expressing (J6) and deficient (J6‐OlaHSD) mice before and after MPTP treatment. Behaviourally, J6 mice were more active but more anxious and had poorer motor endurance. MPTP caused similar dopaminergic loss in both strains; however, J6 mice showed weight loss and greater gliosis in the substantia nigra. In contrast, OlaHSD mice showed increased microglial activation in the caudate putamen. Raman spectroscopy revealed greater protein structural changes in J6 mice post‐MPTP. These results suggest α‐synuclein influences non‐dopaminergic behaviours, glial responses and protein conformation under stress, highlighting its subtle but important neurobiological functions.

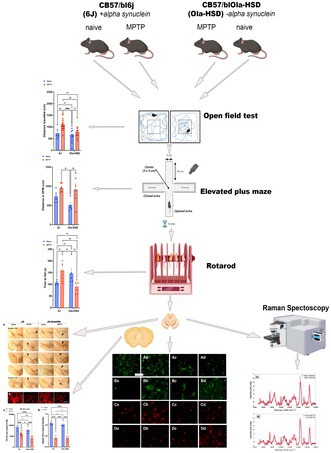

**Abbreviations**
AR: adrenergic receptor
CPu: caudate putamen
DA: dopamine
df: degrees of freedom
GFAP: glial fibrillary acidic protein
IBA‐1: ionised calcium‐binding adapter molecule 1
J6 mice: C57bl/J6 Jackson
MPTP: 1‐methyl‐4‐phenyl‐1,2,3,6‐tetrahydropyridine
OlaHSD: C57bl/6OlaHSD
PD: Parkinson's disease
RMS: Raman micro‐spectroscopy
SN: substantia nigra
SNc: substantia nigra pars compacta
TH: tyrosine hydroxylase
TH‐ir: tyrosine hydroxylase immunoreactivity

## 1 Introduction

α‐Synuclein plays a pivotal role as a primary constituent of Lewy bodies, a major pathological hallmark of Parkinson's disease (PD). Investigating the role of α‐synuclein in PD pathology has been accomplished through various in vivo transgenic murine models. The A53T, A30P and E46K point mutations represent three of several mutations in the α‐synuclein gene associated with familial forms of PD, which are linked to dopaminergic neurodegeneration propagation and disease progression (Goedert et al. 2017). In transgenic mice bearing these point mutations, there is overexpression of the mutant α‐synuclein (Sommer et al. 2000). In these transgenic mice, typical neurodegeneration of the dopaminergic system is not replicated as seen in PD, whereas characteristic motor and non‐motor dysfunctions and responsiveness to dopamine (DA) treatment are observed (Chesselet 2008). Furthermore, these mutations appear to be protective against 1‐methyl‐4‐phenyl‐1,2,3,6‐tetrahydropyridine (MPTP) induced neurotoxicity towards dopaminergic neurons. Conversely, in non‐transgenic animals, intracerebral administration of human A53T/A30P‐mutated α‐synuclein in the rat substantia nigra (SN) (Kirik and Björklund 2003) and intrastriatal administration of preformed α‐synuclein fibrils (Luk et al. 2012; Paumier et al. 2015) led to progressive dopaminergic neurodegeneration.

The role that α‐synuclein plays under normal conditions remains poorly understood. Although the role of α‐synuclein in dopaminergic neurodegeneration has been extensively studied in genetic α‐synuclein deletion (Robertson et al. 2004; Schlüter et al. 2003) or overexpression (Rathke‐Hartlieb et al. 2001) in mice spontaneously lacking α‐synuclein gene (Schlüter et al. 2003), few investigations have focused on the behaviour and differences in inflammatory markers of MPTP‐induced dopaminergic neurodegeneration in the presence or absence of α‐synuclein.

The J6‐OlaHSD C57bl mouse (OlaHSD) strain, also known as C57Bl/6S, bred by Envigo laboratories (now Inotiv), exhibits a spontaneous chromosomal deletion of the *Snca1* gene (Specht and Schoepfer 2001, 2004), which encodes α‐synuclein. Conversely, C57bl/6 Jackson (J6) mice, bred by Charles River laboratories, maintain normal expression of *Snca1*. This distinction between the two C57bl/6 sub‐strains is associated with other neurophysiological variances. For example, OlaHSD displays lower motor impulsivity compared to J6 mice (Peña‐Oliver et al. 2010), and they have reduced levels of dopamine (DA) transporter (DAT) and increased levels of Syn‐3 (a vesicle regulating synapsin) in the striatum compared to J6 and human transgene (SYN120 tg) mice (Faustini et al. 2022). The OlaHSD mice also exhibit significantly lower trabecular bone mass than J6 mice (Liron et al. 2018), and after peripheral nerve damage, OlaHSD mice show higher levels of preserved myelin and macrophage count compared to normal wild‐type α‐synuclein‐expressing controls (Siebert et al. 2010). Also, the presence of endogenous mouse α‐synuclein in 6J mice leads to a higher number of dopaminergic neurons in the SN of mice compared with OlaHSD mice with a spontaneous deletion of the α‐synuclein gene (Garcia‐Reitboeck et al. 2013).

Given the extensive use of 6J mice in MPTP studies and the distinct physiological differences previously described between J6 and OlaHSD mice, we aimed to better understand the role that α‐synuclein may play in murine biology by its exclusion, rather than overexpression. Consequently, more detailed measures such as psychomotor activity and neurochemistry apart from nigrostriatal dopaminergic innervation were investigated.

We attempted to detect and explain any neurochemical differences that might be present using Raman micro‐spectroscopy (RMS). Raman spectroscopy is a non‐destructive and label‐free analytical technique that provides information about the molecular composition and structure of a sample. RMS introduces a distinct approach to gain additional insights into the microscopic samples' chemical composition of the SN of different sub‐strains and represents a valuable addition to the existing molecular detection toolkit. This method leverages the detection of vibrational modes associated with each chemical bond within molecules through the inelastic scattering of light. The inherent wealth of chemical information contained in the Raman spectrum provides valuable insights into biochemical imbalances intrinsic to the lack of expression of α‐synuclein. As a non‐invasive method to characterise chemically complex and spatially heterogeneous samples with a sub‐micrometre spatial resolution, it is routinely used for histological analysis in diverse clinical settings (Mulvaney and Keating 2000), serving diagnostic purposes as well as fundamental biological research.

Therefore, the aim of this study was to compare the differences in behaviour, nigrostriatal dopaminergic integrity and the state of neuroinflammation in both J6 and OlaHSD mice in the absence or presence of a sub‐chronic MPTP treatment. The addition of the RMS offers further insights into the chemical composition of un‐fixed fresh flash frozen tissue sections obtained from the SN of both strains.

## 2 Methods

### 2.1 Animals

All the experiments described complied with ARRIVE guidelines and were carried out in accordance with the UK Animals (Scientific Procedures) Act 1986 and EU directive 2010/63/EU for animal experimentation. Prior to the initiation of the studies, all procedures and protocols were approved by the University of Hertfordshire Animal Welfare and Ethical Review Board (AWERB) and were carried out under the UK Home Office‐approved Project Licence PD7255B4. Male mice, aged 8–10 weeks (19–21 g) were purchased from either Charles River (J6, RRID:MGI:5818271) or Envigo Laboratories (now Inotiv, UK) (J6‐OlaHSD, RRID:IMSR_ENV:HSD‐057). Since the C57BL/6JOlaHSd (OlaHSD), also known as C57Bl/6S, are subline carriers of spontaneous α‐synuclein deletion, they were not readily available and could not be sourced from Charles River. Mice were housed in groups of 3 and those from different labs were not housed together. Access to food and water was ad libitum, room temperature was maintained at 22°C–24°C and the light cycle was automated at 12 h (light):12 h (dark). All experiments were carried out under an ambient light intensity of 100 Lux between 09:00 and 14:00 h.

### 2.2 MPTP Treatment

The two C57bl/6 sub‐strains, J6 (*n* = 24) and OlaHSD (*n* = 24) were split into two groups for treatment with either vehicle or MPTP, totalling four groups: J6 vehicle, J6 MPTP, OlaHSD vehicle and OlaHSD MPTP. The sample sizes were chosen based on previous behavioural investigations in mice (Powell et al. 2022). MPTP administration acutely induces tachycardia but also has an acute effect on adrenergic receptors (AR) in cardiac and vascular tissue, resulting in vasoconstriction and hypothermia, which can lead to heart failure and death of the animal (Fuller et al. 1984; Liu et al. 2020; Przedborski et al. 2004). We initially encountered approximately 3–5 deaths mainly in the 6J group but intervened early to prevent suffering by culling animals that were moribund. This did not exceed 4 in the MPTP group. However, subsequently, these deleterious acute effects were avoided if dosing of MPTP was incremental, thereby allowing for de‐sensitisation of vascular and peripheral ARs. We employed this protocol, which subsequently led to all MPTP‐treated mice surviving a sub‐chronic MPTP dosing regimen (s.c.), single dose per day. For ease of calculations, the dose of MPTP was first calculated as total salt content but adjusted to MPTP free base in Table 1. Furthermore, because of the risk of contamination, MPTP‐treated mice had to be separated from non‐MPTP‐treated animals. Therefore, no blinding was performed during data acquisition. However, for assessment of behaviour, animals were arbitrarily assigned in the following manner to blocks: (A) vehicle J6, (B) MPTP J6, (C) vehicle OlaHSD, (D) MPTP OlaHSD.

**TABLE 1:** Timeline of MPTP treatment and trial series of open field (OFT), elevated plus maze (EPM) and rotarod (RR). Mice were culled at Day 30. MPTP doses were initially given as total salts but presented as freebase doses in the table.

Day	1	2	3	6	7	8	9	10	15	16	17	27	28	29	30
Trials	OFT‐1	EPM‐1	RR‐1						OFT‐2	EPM‐2	RR‐2	OFT‐3	EPM‐3	RR‐3	Cull
MPTP freebase dose (mg/kg)	—	—	—	11.11	21.37	27.35	29.91	29.91	—	—	—	—	—	—	—

### 2.3 Apparatus

All open field and elevated plus maze (EPM) behavioural assessments were carried out using a Basler infrared camera (Basler AG, Germany), infrared light boxes and analysed with the Noldus EthovisionXT (RRID:SCR_000441) tracking software (Tracksys, Nottingham, UK). See our previous paper (Powell et al. 2022) for a detailed description of the maze apparatus. Prior to any drug or vehicle treatment, the basal behaviour between each mouse sub‐strain was assessed for differences in three different apparatuses for the evaluation of kinetic and affective behaviours (see Table 1 for timeline of tests).

### 2.4 Open Field

To determine the extent of thigmotaxis, a 30 cm^2^ central zone that sat inside the centre of the arena was digitally created with the Ethovision‐XT software, thus allowing a 5 cm wide channel to run around the perimeter of the OFT. The following parameters were measured electronically using the Ethovision‐XT software: total distance travelled (cm); distance travelled, duration (time spent in s) and mean velocity (cm/s) in the centre area or perimeter and number of entries into centre.

### 2.5 Elevated Plus Maze

Mice were placed onto the centre square of the EPM facing towards the open arms. To determine when mice crossed into the different arms of the EPM, five separate zones were digitally created: two for the closed arms, two for the open arms and one for the centre square where all four arms cross. Duration (time spent in s) in the open arms, closed arms and centre square, as well as entries into the open arms and closed arms were calculated using the Ethovision‐XT software.

### 2.6 Rotarod

Mice were trained on the five compartment Rotarod (Ugo‐Basile 47650, Gemonio, Italy) to become familiarised with the apparatus before any motor activity was recorded. Five mice at a time were arbitrarily selected from each group and were subjected to the Rotarod trial. Each test consisted of three trials: one trial in the morning (which was considered as the training trial) and two experimental trials in the afternoon, with each trial separated by 1 h (Ayton et al. 2013). Mice were placed on the Rotarod one by one at a speed of 4 rpm. Mice traversed the Rotarod with the speed increasing incrementally every 2 s by 1 rpm, culminating in 35 rpm and continued to maintain that speed until all mice had fallen off. The duration that mice stayed on the Rotarod was recorded, and a mean value of the two afternoon trials was used as the final result.

### 2.7 Immunohistochemistry

Mice were culled by exposure to CO_2_ according to NIH guidelines (CO_2_ flow rate: 5–6 L/min) in a Vet‐Tech medium red chamber (Vet‐Tech, UK), transcardially perfused with ice‐cold phosphate buffered saline (PBS), decapitated, brains fixed in formalin (10% buffered) and prepared accordingly for immunohistochemical (IHC) analyses. Brains were sectioned coronally at 30 μm thickness and analysed for avidin‐biotin visible light IHC for detection of nigral tyrosine hydroxylase (TH)‐immunoreactive (ir) neurons and stereology. Immunofluorescence (IF) immunohistochemistry was carried out to detect TH‐ir axon terminals in the striatum (caudate putamen, CPu), astrocytes and microglia using sheep tyrosine hydroxylase (TH), 1:500 (Thermo Fisher Scientific Cat# PA1‐4679, RRID:AB_561880), chicken glial fibrillary associated protein (GFAP) 1:1000 (Thermo Fisher Scientific Cat# PA1‐10004, RRID:AB_1074620) and rabbit ionised calcium‐binding adapter molecule 1 (Iba1) 1:500 (Abcam Cat# ab178847, RRID:AB_2832244). Free‐floating sections were washed in PBS (1×), triton X‐100 (0.1%), non‐specific binding was inhibited with non‐animal protein blocker (Vector) and incubated overnight in primary antibody at 4°C. The following day, sections were washed and incubated in secondary antibody for 1 h at room temp (20°C–22°C). The relevant secondary antibodies were tagged with AlexaFluor 594 (donkey anti‐sheep Thermo Fisher Scientific Cat# A‐11016, RRID:AB_2534083; goat anti‐rabbit Thermo Fisher Scientific Cat# A‐11012, RRID:AB_2534079) and AlexaFluor 488 (goat anti‐chicken Thermo Fisher Scientific Cat# A‐11039, RRID:AB_2534096). For avidin‐biotin reactions, 3,3‐diaminobenzidine (DAB) was used as a chromagen (DAB kit from Vector). Tissue was mounted on slides, and images were taken on a Zeiss Axiophot light microscope and photographed with a Zeiss AxioCam SE.

Cell counting in the SN was performed using stereological quantification (Ip et al. 2017). From within the boundaries of the nigrostriatal bundle (~Bregma −2.50) and the caudal portion of the SNc (~Bregma −3.90), five TH‐ir sections, 300 μm apart (every 10th section) were observed and cells were digitally identified and counted using ImageJ. The following calculation was used to achieve the final estimate of TH‐ir neurons.

$$N=\Sigma_{i}^{5}=1\left(A_{i}\times \mathrm{ssf}\right)\times \mathrm{mcf}$$

The estimated number of cells (*N*) has been derived where Σ is the sum of sections (*i*) ranging from 1 to 5 ($\Sigma_{i}^{5}$). ssf (= 10) is the sampling fraction, which is one section, and *Ai* is the area of that section analysed every 10 sections. mcf is the missing cell fraction of uncounted cells, cells laying on their side or layered on top of each other through the *Z* axis = 1.33.

### 2.8 Integrated Optical Density Measurement

Red fluorescence images were converted into 8‐bit grayscale images and compared against a grayscale stepladder from which a calibration curve was obtained. For fluorescence images, this calibration curve was reversed (an ascending rightward shift instead of descending for visible light avidin‐biotin complex immunohistochemistry using DAB) (Iravani et al. 2005). To assess the extent of nerve terminal loss in the CPu, integrated optical density of striatal sections (3–4 striatal sections per animal) was processed for fluorescence TH‐ir. To determine the extent of striatal TH‐ir following MPTP or vehicle, the extent of TH‐immunostaining was assessed from areas within the CPu using a small digital measuring probe (approx. 200 × 200 μm at ×20 magnification) at 5–8 sites. Variations in staining intensity within the dorsal CPu subregions (Iravani et al. 2006) were compared in different animal groups using ImageJ image analysis software.

### 2.9 Raman Spectroscopy: Setup, Data Collection and Analysis

Raman spectroscopy measurements were performed using a custom‐built near‐infrared micro‐Raman setup. This setup included an inverted optical microscope (Ti‐Eclipse, Nikon, Japan) equipped with a water‐immersion objective (63×/NA 1.0) (Zeiss, Germany). The excitation source was a 710 nm Gaussian‐beam Ti laser (Spectra‐Physics) delivering approximately 170 mW at the sample. The setup also featured a motorised XY stage (Prior Instruments, Canada).

The laser was filtered outside the cavity to clean spurious background. The laser beam was expanded to fill the back aperture of the objective, enabling the focus of the laser beam to a diffraction‐limited spot on the sample. Inelastic scattered Raman photons were collected by the same microscope objective in a backscattering configuration. The optical microscope was connected via a focusing lens and a 50 μm diameter optical fibre to a Newport Oriel 77200 spectrograph, coupled to a deep‐cooled Near IR detector (iDus BR‐DD 420, Andor, Belfast, UK).

Tissue sections, each 40 μm thick, were mounted on custom‐designed microscope slides (25 mm diameter and 15 mm height) with CaF_2_ coverslips (0.17 mm thick, 20 mm diameter; Crystran, UK) to facilitate the acquisition of Raman spectra using an inverted optical configuration. Each section was raster‐scanned over an area of 100 × 100 μm with a step size of 2 μm and a dwell time of 1 s per pixel. The spectral resolution in the 400–1800 cm^−1^ region was approximately 1.5 cm^−1^, with an acquisition time of 1 s per position.

After acquisition, each individual spectrum underwent pre‐processing for cosmic ray removal and background subtraction. Raman shift wave‐number axis was calibrated for each experiment with the use of a standard Tylenol sample. The peak deconvolution analysis, presented in Sections 3 and 4, utilised curve fitting tools in Matlab (MathWorks, USA).

### 2.10 Data Analysis and Statistics

Each parameter for changes in the open field activity test (OFT) and the EPM was analysed using an un‐paired *t*‐test where only the behavioural measures from the two mouse genotypes were compared. For comparison of behaviours between the two genotypes, an un‐paired, one‐tailed Student's *t*‐test was used with degrees of freedom presented as df. For comparison of behavioural tasks (open field and elevated plus maze) and analysis of immunohistochemical data in vehicle or MPTP‐treated 6J and OlaHSD animals, a two‐way analysis of variance (ANOVA) after vehicle or MPTP treatment against genotype was performed comparing mouse strains. For comparison of weight, genotype and treatments, a three‐way ANOVA was used. When a statistically significant difference was observed overall, the data were further analysed using Tukey's post hoc test. Differences between group means were considered statistically significant when *p* < 0.05. All datasets were assessed for normal distribution using graphical methods, specifically Quantile–Quantile (*Q*–*Q*) plots, and statistically through the D'Agostino‐Pearson omnibus (*K* ^2^) test. The distribution met the criteria for normality at a significance level of *α* = 0.05 or greater. No outlier tests were conducted. Statistical analyses were performed using GraphPad Prism version 10.4 (RRID:SCR_002798).

## 3 Results

### 3.1 Body Weight and the Effects MPTP

**FIGURE 1:**
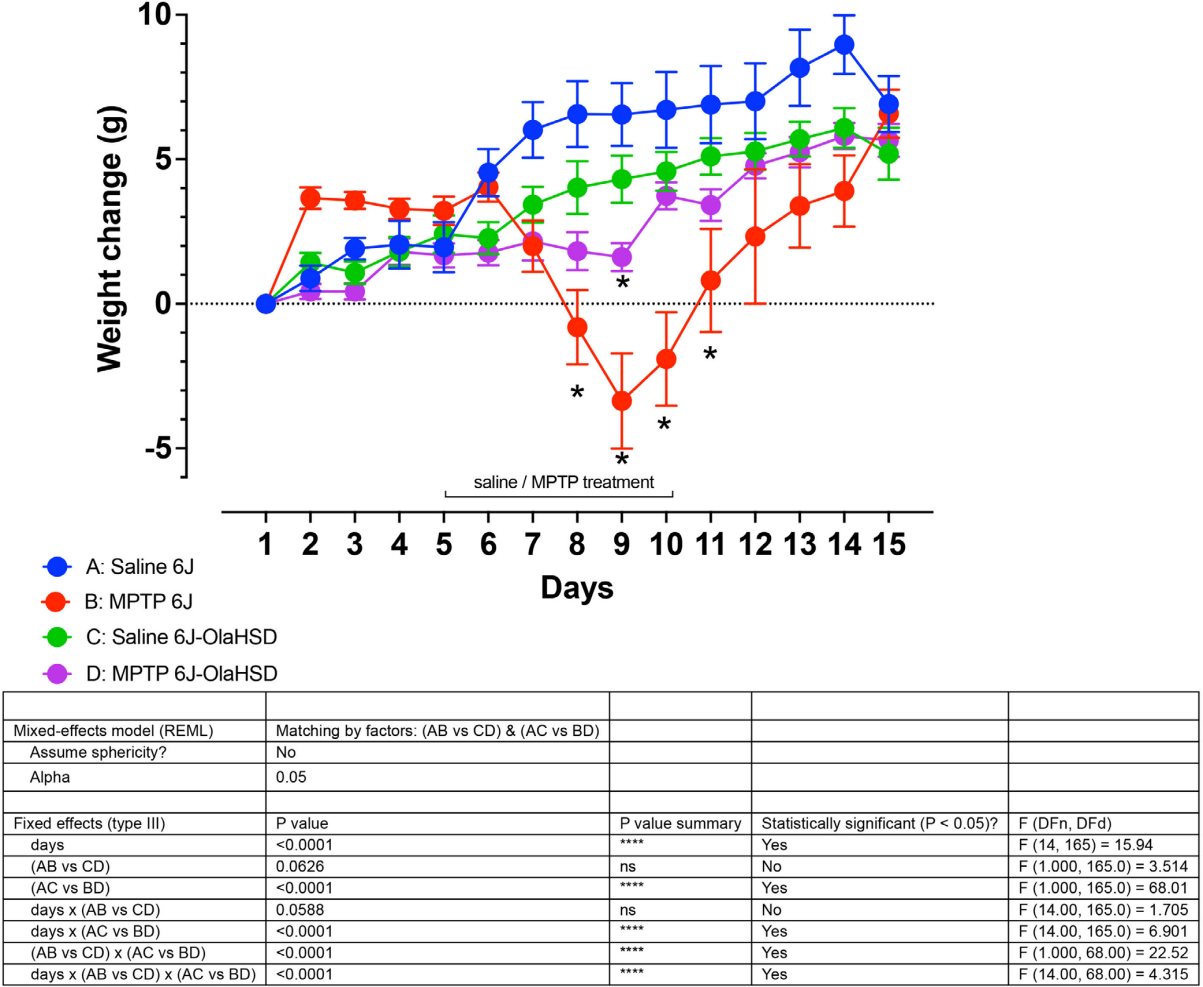
Changes in body weights during treatment. Body weight following different treatments and in different genotypes were assigned to groups A–D and were compared using a three‐way ANOVA followed by Tukey's post hoc test for differences. Differences were compared using mixed‐effect analysis, *n* = 6–12, where *p < 0.05. Four days after MPTP treatment, there was a significant difference between groups B and D.

There was a daily increase of body weight in all groups. In the MPTP treatment groups B (6J) and D (OlaHSD), there was weight loss which peaked at Day 4 of sub‐chronic MPTP treatment. However, in 6J group B, weight loss was most striking (Figure 1). A three‐way ANOVA which compared treatment, followed by a Tukey's post hoc test, revealed a statistically significant difference between 6J and OlaHSD following MPTP treatment. Overall, there were no significant differences between the body weights of 6J (AB) and OlaHSD (CD) phenotypes during the study. The passage of time also did not significantly affect weight in the two strains, but MPTP treatment significantly affected body weight (AC vs. BD) so did the passage of time in the MPTP‐treated group. While a significant reduction in body weight occurred in the MPTP‐treated J6 mice, the changes in the body weight of OlaHSD remained unchanged compared to vehicle‐treated controls. There were five animals removed from group B, the 6J MPTP group, due to acute MPTP toxicity by culling by *Schedule 1* to prevent the unnecessary suffering of the affected animals.

### 3.2 Open Field Test (OFT)

**FIGURE 2:**
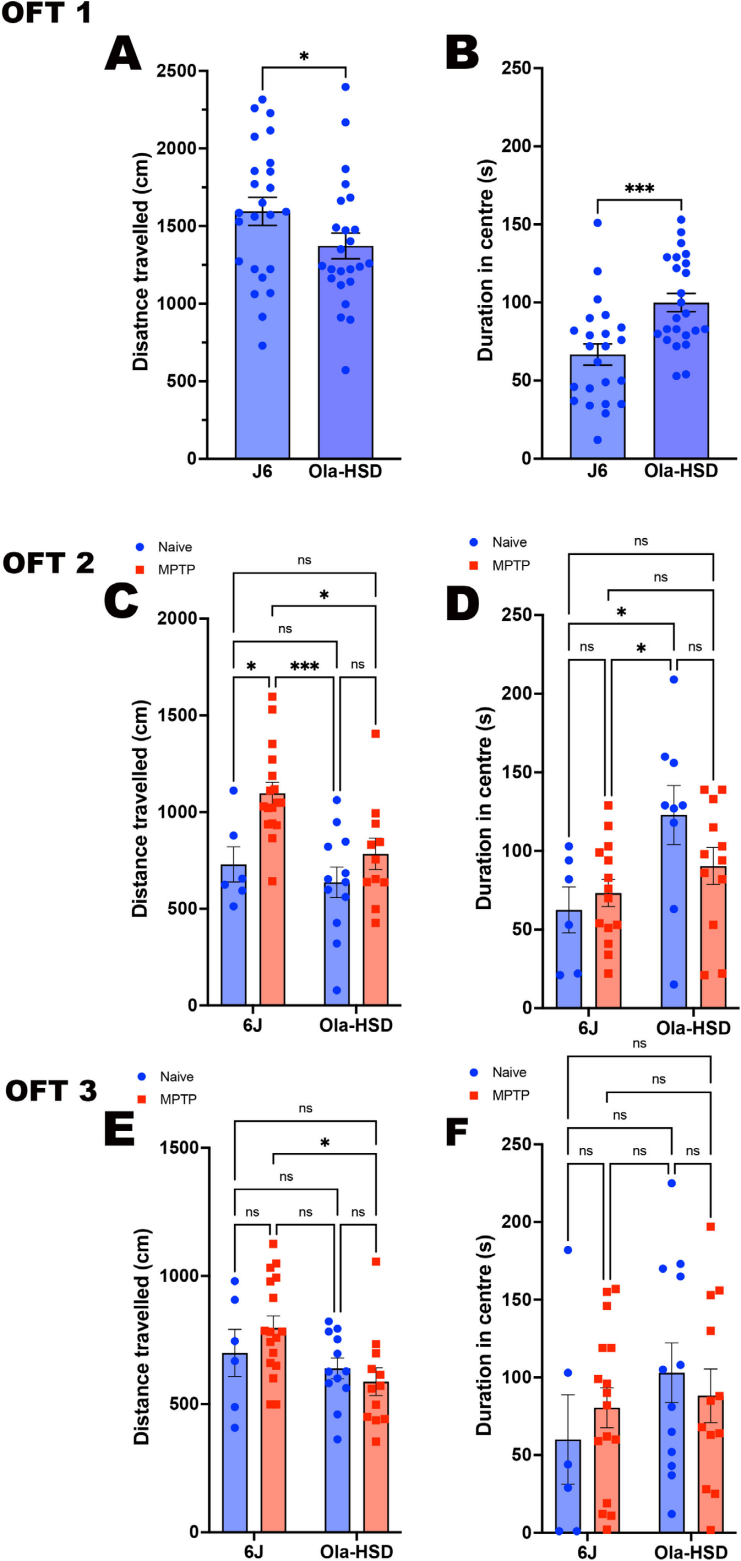
Locomotor profile of 6J and OlaHSD mice for the distance travelled and time duration spent in the centre of the arena following MPTP or vehicle treatment at different sessions in open field test (OFT). Five days prior to treatment with vehicle or MPTP, the basal distance travelled (A) and duration of time spent in the centre of arena (B) showed that OlaHSD mice travelled less (*p = 0.0379; df = 45, one‐tailed Student's *t*‐test) but spent significantly more time in the centre of the arena (***p = 0.003, df = 44, one‐tailed Student's *t*‐test). At OFT2 there was a significant difference overall distance travelled (C) by the different mice genotypes, [*F*(1, 42) = 6.403, *p* = 0.0152], and the MPTP‐treated J6 mice travelled significantly greater distance compared to all other groups [*F*(1, 42) = 10.32, *p* = 0.0025]. Duration in the centre of the arena (D) was only significantly different between genotypes; untreated OlaHSD spending significantly greater time in the centre of arena [*F*(1, 37) = 8.102, *p* = 0.0072]. This genotype difference for duration in the centre was maintained in the OFT3 session [*F*(1, 42) = 5.572, *p* = 0.0229] (E, F). However, there was no significant difference between treatments on either distance travelled or duration of time spent in the centre of the arena. A Tukey's multiple comparison post hoc test was used in OFT 2 and OFT 3; *p < 0.05, ***p < 0.0001, error bars denote SEM.

Five days prior to treatment with vehicle or MPTP, during the course of 5‐min exposure to open field test (OFT 1, Figure 1A,B), the basal distance travelled and duration of time spent in the centre of the arena showed that OlaHSD mice travelled significantly less but spent significantly more time in the centre of the arena. In the OFT 2 session (Figure 2C,D), MPTP‐treated J6 mice travelled a significantly greater distance compared to all other groups, whereas OlaHSD vehicle‐treated mice spent significantly longer time in the centre of the arena compared to all other groups (Figure 2D). However, after MPTP, the differences between groups in time spent in the centre of the arena were no longer apparent. In the OFT 3 (Figure 2E,F) session, the behavioural trends were like OFT 2, but the differences were not as pronounced and were largely statistically insignificant. However, there was a significantly greater reduction only in distance travelled in MPTP‐treated OlaHSD compared to MPTP‐treated 6J (Figure 2E). The differences between time spent in the centre of the arena by J6 and OlaHSD vehicle‐treated or MPTP‐treated were no longer significant (Figure 2F).

### 3.3 Elevated Plus Maze

**FIGURE 3:**
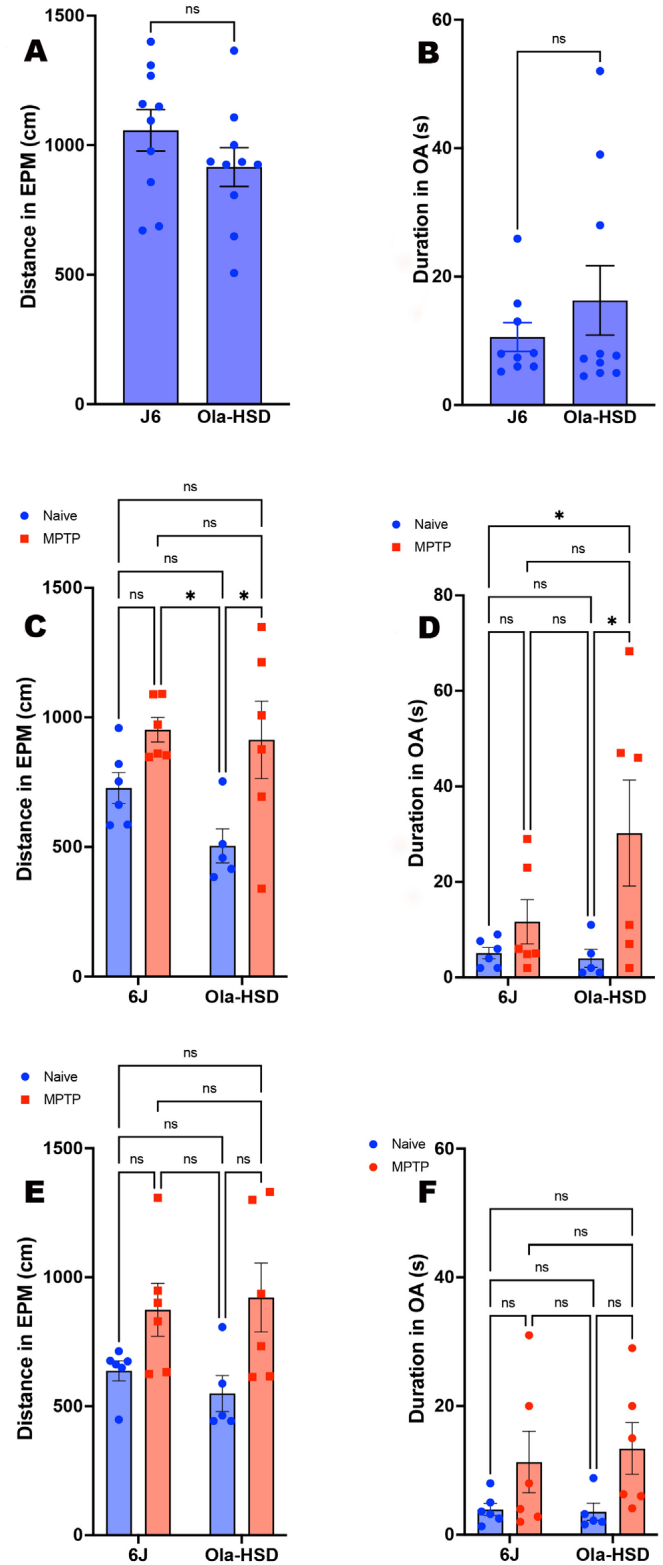
Elevated plus maze profile of 6J and OlaHSD mice for the distance travelled in the elevated plus maze (EPM) and time duration spent in the open arm (OA) following MPTP or vehicle treatment at different sessions. There was no significant difference between 6J and OlaHSD in the distance travelled in the EPM or the duration spent in OA 4 days prior to vehicle or MPTP treatment (A, *p* = 0.181, df = 17 un‐paired Student's one‐tailed *t*‐test) and (B, *p* = 0.105, df = 18 un‐paired Student's one‐tailed *t*‐test) respectively. Six days following vehicle or MPTP treatment, the distance moved and the time spent in the OA in 6J and OlaHSD genotypes (EPM 2; C, D), were significantly different with regards to MPTP in the distance moved (C) [*F*(1, 19) = 11.74, *p* = 0.0028] and duration of movement in the OA (D) [*F*(1, 19) = 6.556, *p* = 0.0191]. Eighteen days post vehicle or MPTP treatment (EPM 3; E, F), a similar pattern was seen for distance moved in EPM (E) [*F*(1, 19) = 10.13, *p* = 0.0049] and duration in OA (F) [*F*(1, 19) = 6.529, *p* = 0.0193]. A two‐way ANOVA followed by Tukey's multiple comparison post hoc was used to compare the effect of MPTP treatment and genotype on locomotor and behavioural responses in panels C–F. *p < 0.05, error bars denote SEM.

There were no significant differences 4 days prior to vehicle or MPTP treatment, distance moved in EPM (Figure 3A) and the time spent in open arm (OA) (Figure 3B). In the EPM2, MPTP treated only the OlaHSD mice travelled further compared to both vehicle groups (Figure 3C), and only MPTP‐treated OlaHSD mice spent significantly longer in the open arms of the EPM compared to all other groups (Figure 3D). The EPM 3 followed a similar trend to EPM 2, whereby both MPTP groups had greater locomotor activity, but this difference did not reach statistical significance (Figure 3E), and distance in the open arms was greater in MPTP‐treated 6J and OlaHSD mice but non‐significantly (Figure 3F).

### 3.4 Test of Motor Coordination Using Rotarod (RR)

**FIGURE 4:**
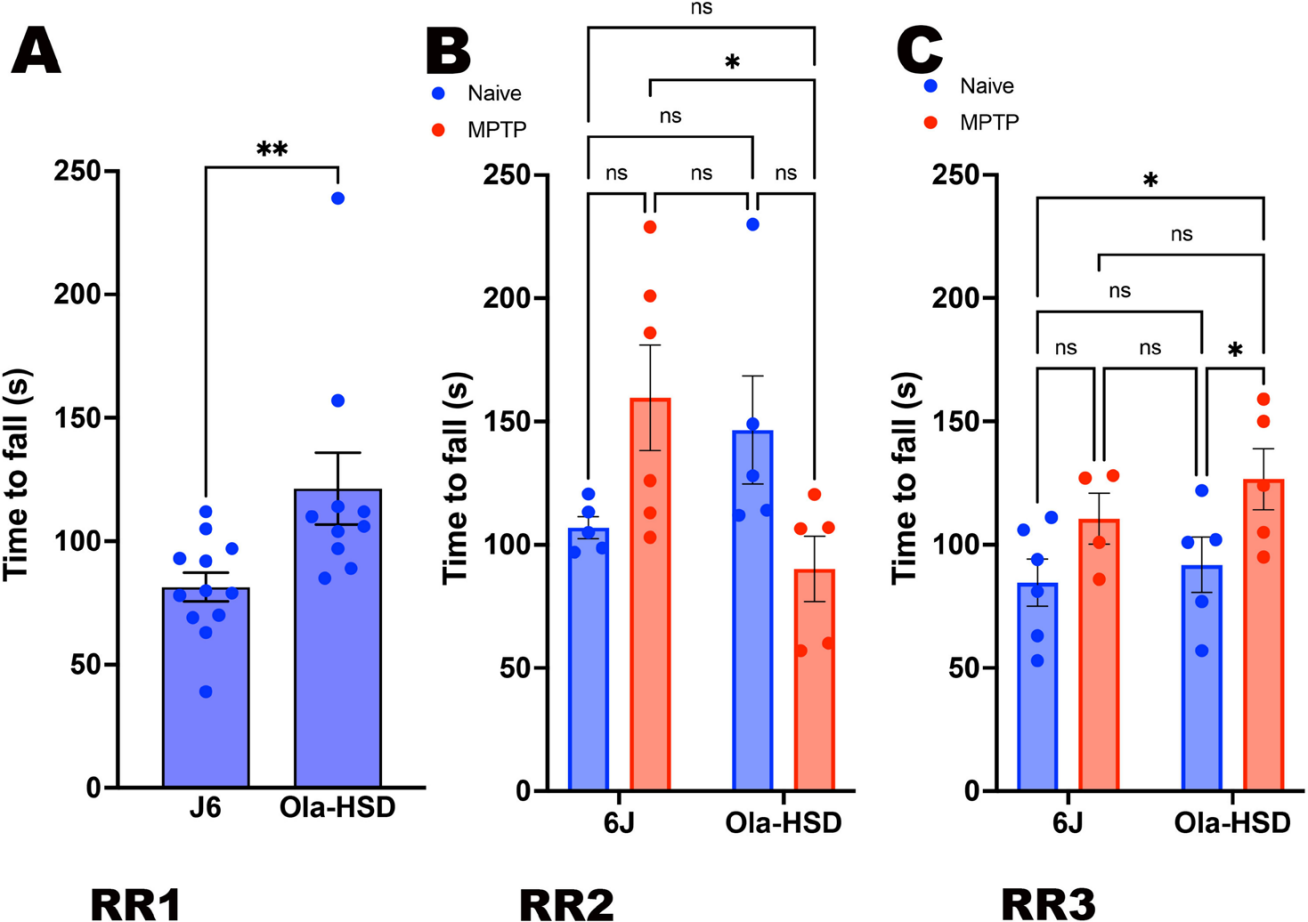
Locomotor coordination using rotarod test (RR) in J6 and OlaHSD mice treated with vehicle or vehicle. Prior to vehicle or MPTP treatment, OlaHSD mice had a significantly greater locomotor coordination than 6J mice (**p = 0.0065, df = 20, one‐tailed Student's un‐paired *t*‐test; A). Seven days following vehicle or MPTP treatment (RR2) the difference in coordination between the vehicle‐treated genotypes were lost [*F*(1, 16) = 1.103, *p* = 0.3093] but MPTP‐treated OlaHSD animals were significantly less coordinated [F(1, 16) = 7.510, *p=0.0145] (B). Nineteen days after vehicle or MPTP treatment the RR3 data showed that all groups were less coordinated (C) and no differences were seen in time to fall in genotype and treatment [*F*(1, 15) = 0.0237, *p* = 0.883]. Comparisons were made using a two‐way ANOVA followed by Tukey's multiple comparison post hoc test, error bars denote SEM.

The baseline time spent on the rotarod (RR1) by OlaHSD was significantly longer compared to J6 (Figure 4A). In RR2, no differences were observed in vehicle‐treated animal groups, but the MPTP‐treated OlaHSD animals were less coordinated (Figure 4B). In RR3, no statistically significant difference between J6 and OlaHSD was detected (Figure 4C).

### 3.5 The Effects of MPTP Lesion on Tyrosine Hydroxylase Immunoreactivity

**FIGURE 5:**
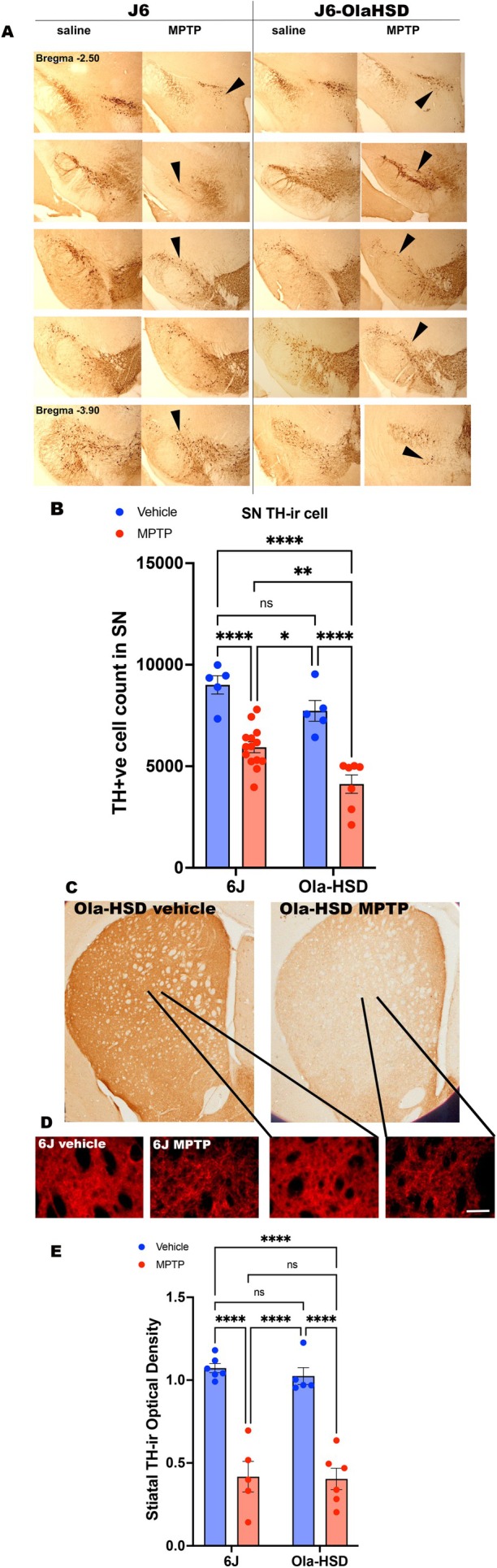
Representative images of TH‐ir stained SN and CPu in vehicle and MPTP‐treated J6 and OlaHSD mice. Nigral sections were taken from Bregma −2.50 at the nigrostriatal bundle through to Bregma −3.90 at the caudal portion of the SNc (A). Following MPTP treatment the number of TH‐ir neurons in both genotypes were significantly reduced [*F*(1, 17) = 63.2, *p* < 0.0001] but the response of the genotypes to MPTP were significantly different [*F*(1, 17) = 13.46, *p* = 0.0011] with more TH‐ir loss in the OlaHSD genotype (B). Multiple comparison with Tukey's *post hoc* test indicated MPTP treatment highly significantly reduced the number of TH‐ir neurons (6J vehicle vs. MPTP, *p* < 0.0001, and OlaHSD vehicle vs. OlaHSD MPTP, *p* < 0.0001; B). There also was a greater loss of TH‐ir neurons in the OlaHSD genotype compared J6 (6J MPTP vs. OlaHSD MPTP, *p* = 0.0059). The number of TH‐ir neurons counted stereologically in each vehicle‐treated genotype were not significantly different (*p* = 0.206). Low magnification images of striatal TH‐immunoreactivity in OlaHSD mice following systemic vehicle (saline) or MPTP treatment is shown panel C. Representative fluorescence TH‐ir at ×20 for each genotype and treatment group is shown in panel D. Similar to panel C, there was a corresponding reduction of fluorescence staining following MPTP treatment (D). The mean integrated optical density of TH‐ir fibres in the CPu was highly significantly reduced following MPTP in both genotypes (E) [*F*(1, 17) = 38.0, ****p < 0.0001] but there were no significant differences in the TH‐ir in the vehicles treated genotypes [*F*(1, 19) = 0.260, *p* = 0.615]. Two‐way ANOVA followed by Tukey's multiple comparison post hoc test indicated ****p < 0.0001, **p < 0.01 and *p < 0.05. *n* = 6–12, error bars denote SEM. The arrows in the panel A indicate TH‐ir neurons. Scale bar in panel D represents 50 μm.

Vehicle‐treated mice belonging to 6J and MPTP had a very similar number of TH‐ir neurons and TH‐ir nerve terminals (Figure 5). MPTP treatment caused the loss of TH‐ir neurons in the SN of both 6J and OlaHSD (Figure 5A). Stereological estimates of nigral TH‐ir cell count (Figure 5B) showed that MPTP treatment in both genotypes revealed a highly significant reduction in the number of TH‐ir neurons. In the SNc of OlaHSD mice, the loss of TH‐ir neurons was approximately 40%, whereas in the J6 mice, a 34% loss occurred. This is highlighted by the fact that there was no significant difference between the two vehicle groups. However, there was a significant difference between the two MPTP groups, with OlaHSD having markedly fewer TH+ neurons than J6 following MPTP treatment.

Striatal TH‐ir in J6 and OlaHsd were equally reduced by MPTP (Figure 5C,D) compared to vehicle treatment, which showed that MPTP caused a greater level of loss of TH‐ir nerve terminals. Loss of TH‐ir fibres in the CPu was similar for both sub‐strains administered with MPTP (−63% compared to vehicle‐treated mice) (Figure 5E).

### 3.6 Reactive Gliosis

**FIGURE 6:**
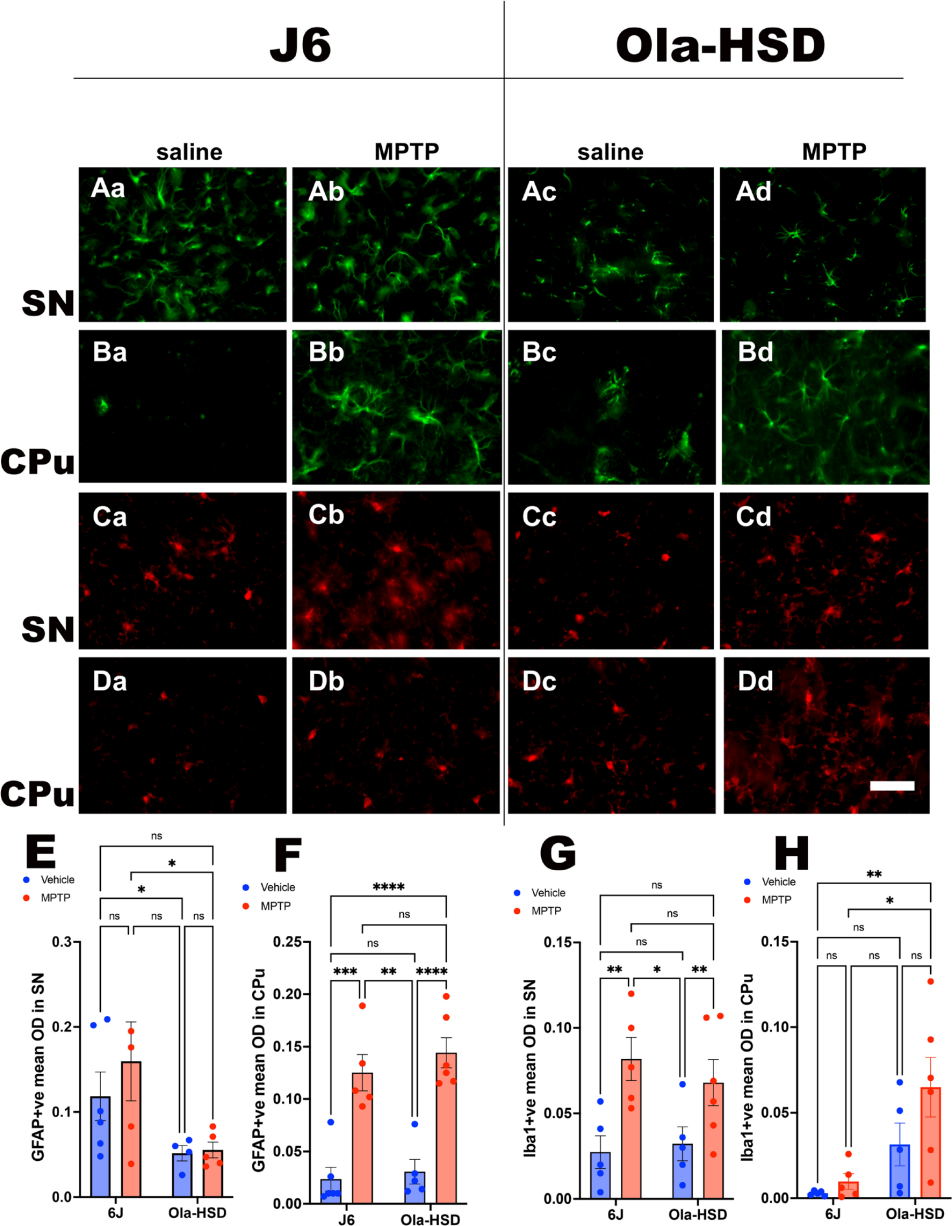
Representative images of GFAP‐ir immunofluorescence (green) in the saline‐ or MPTP‐treated mice SN (Aa–Ad) and CPu (Ba–Bd) are shown. Panels Aa, Ab, Ba and Bb represent 6J and Ac, Ad, Bc and Bd are images of OlaHSD mice. The Iba‐1‐ir representative images (red) showing saline‐ or MPTP‐treated SN (Ca–Cd) and the CPu (Da–Dd) of 6J and OlaHSD mice. Mean optical density of GFAP‐ir in the SN (E) of the two mouse strains were statistically different [*F*(1, 17) = 8.568 *p* = 0.0094] but MPTP treatment did results in a significant change [*F*(1, 17) = 0.696, *p* = 0.415]. Tukey's multiple comparison post hoc test indicated that a significant difference between the optical density of GFAP‐ir following MPTP treatment between 6J and OlaHSD mice (*p* = 0.0424). In the CPu (F), no statistical difference in GFAP‐ir was observed between genotypes [*F*(1, 19) = 0.930, *p* = 0.346] but MPTP treatment significantly increased GFAP‐ir in both genotypes [*F*(1, 19) = 63.48 *p* < 0.0001]. In the SN, there was no statistically significant difference between Iba‐1‐ir (G) in the vehicle‐treated genotypes [*F*(1, 18) = 0.175, *p* = 0.680] but MPTP treatment lead to significant increase in Iba‐1‐ir [*F*(1, 18) = 14.60, *p* = 0.0013]. However, there was statistically significant difference between Iba‐1‐ir in the CPu (H) of vehicle‐treated genotypes [*F*(1, 18) = 12.47, *p* = 0.0024]. Treatment with MPTP did not have an overall significant effect [*F*(1, 18) = 2.990, *p* = 0.1009]. Two‐way ANOVA followed by Tukey's multiple comparison post hoc test indicated ****p < 0.0001, **p < 0.01 and *p < 0.05. *n* = 6–12, error bars denote SEM. The arrows in the panel A indicate TH‐ir neurons. Scale bar in panel D represents 50 μm.

Reactive microgliosis and astrogliosis were analysed in the CPu and SN from all four groups. While there were no statistical differences between vehicle or MPTP treatment in the SN of both genotypes, the basal levels of GFAP‐ir reactive astroglia were statistically greater in the SN of J6 mice compared to OlaHSD mice, with or without MPTP treatment (Figure 6Aa–Ad,E). Assessment of GFAP‐ir in the CPu revealed a marked increase in astrocytosis following MPTP treatment in both genotypes. Compared to vehicle‐treated mice, MPTP mice showed a marked and statistically significant increase in astrocytosis (Figure 6Ba–Bd,F). Reactive microgliosis was highly increased in the J6 and OlaHSD strains after MPTP treatment (Figure 6Ca–Cd,G). However, in the CPu, the Iba‐1‐ir was statistically increased only in the OlaHSD strain following MPTP treatment [*F*(1, 17) = 12.27, *p* = 0.0027] (Figure 6Da–Dd,H).

### 3.7 Raman Spectroscopy

**FIGURE 7:**
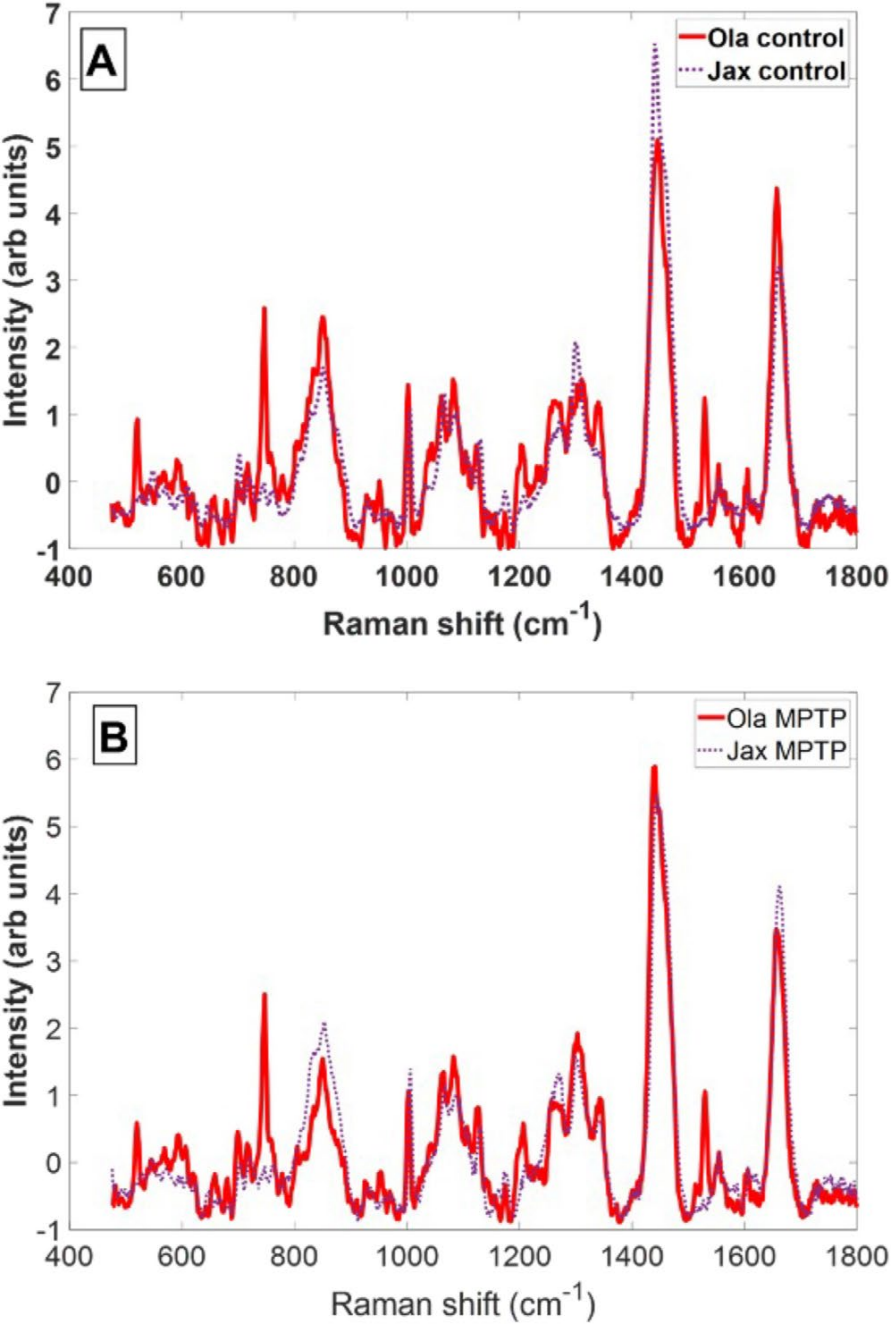
Average Raman spectra of maps covering substantia nigra pars compacta (SNc) from drug‐naïve (A) and MPTP (B) treated J6 and OlaHSD mice brain sections scanned using micro‐Raman.

Raman spectra presented were obtained from the SN region of the mouse brain. The area of scanning was chosen based on the online mouse brain atlas (https://labs.gaidi.ca/mouse‐brain‐atlas/). The data analysis uses the average spectra contained in each of the raster‐scanned regions. The spectra presented in Figure 7 are an average over 2500 spectra collected in the raster scanning mode area (100 μm × 100 μm) on the un‐fixed nigral tissue sections. Raman spectra recorded in the fingerprint region from 400 to 1800 cm^−1^ are dominated by the presence of protein peaks, carbohydrates and lipids. Easily identifiable peaks are at 519–520 cm^−1^ where the tertiary structure of proteins is assigned to disulphide bonds (Rygula et al. 2013), phenylalanine assigned to around 1001 cm^−1^. Amide III region from 1170 to 1380 cm^−1^, carbo hydrogen CH region between 1380 and 1500 cm^−1^ and amide I region 1630–1700 cm^−1^ (Rygula et al. 2013). Strong individual peaks were observed around 747 cm^−1^ (Okada et al. 2011) and 1530 cm^−1^ and are present only in OlaHSD sub‐strains but were not visible in the Raman spectra of J6 sub‐strains in either control or MPTP‐treated animals. The peak at 747 cm^−1^ is likely to indicate the presence of cytochrome *c* (Okada et al. 2011). The peak observed around 1530 cm^−1^ in OlaHSD samples is attributed to deoxyguanosine triphosphate (dGTP) (Pezzotti 2021).

## 4 Discussion

### 4.1 Behaviour and Motor Activity

We investigated the behavioural, neuro‐histochemical and molecular spectral properties of two different C57bl/6 laboratory mouse sub‐strains, J6 and OlaHSD. The initial behavioural baseline analyses showed distinct differences between the two groups. J6 mice have a greater locomotor activity, travelling further than OlaHSD mice over 5 min in the open field, which also corresponds with greater velocity, but J6 mice spend a greater amount of time in the perimeter of the arena compared to their OlaHSD counterparts, indicative of J6 mice avoiding the open space through increased thigmotaxis. This confirmed our previous finding (Powell et al. 2022) and this suggests that J6 are more prone to anxiety than the OlaHSD. This is a trend that both vehicle‐treated groups retained in OFT 2 and OFT 3, suggesting a strong phenotypic set of behaviours that do not wane even after maze habituation. MPTP treatment induced locomotor activity in OFT 2 and OFT 3, EPM 2 and EPM 3, compared to both vehicle groups. Given the dosing regimen employed in this experiment, this is a commonly observed response in MPTP‐treated C57bl/6 mice. The dosing regimen appears to be key in whether MPTP treatment leads to an increase or decrease in locomotor activity; for example, acute MPTP treatment causes reductions in locomotor activity, whereas chronic and sub‐chronic MPTP treatments almost always lead to a greater level of locomotor activity (Fredriksson et al. 1990; Colotla et al. 1990; Chia et al. 1999; Sedelis et al. 2000). The maze arena is also key for teasing out differences between locomotor responses, as it is only MPTP‐treated J6 mice that have an increase in locomotor drive in the open field and not MPTP‐treated OlaHSD mice, but in the plus maze this MPTP‐induced locomotor response is equally seen in both strains of mice in EPM 2 and EPM 3. Interestingly, the baseline Rotarod (RR1) test shows that it is the OlaHSD mice that endure the longest before falling off. This finding contradicts the present locomotor response findings, at least if the RR is solely considered a test of motor function. When considered an apparatus of perseverance, similar to the forced swim test (an assay designed to measure behavioural despair), it is also testing the will of the animal, as well as motor function and balance. Interestingly, Rotarod‐trained mice are shown to have a greater frontal cortex volume compared to un‐trained controls (Scholtz et al. 2015), a phenomenon that may not occur in equal measure between J6 and OlaHSD mice.

### 4.2 MPTP Lesion and Reactive Gliosis

Our study shows a greater loss of TH‐ir DA neurons in the SN of OlaHSD mice (−40%) compared to J6 mice (−34%), potentially suggesting that J6 DA neurons are better protected towards MPTP toxicity. This aligns with Schlüter et al. (2003), who reported that the spontaneous deletion of α‐synuclein in OlaHSD mice does not provide greater protection to DA neurons in the SN following MPTP treatment compared to wild‐type C57bl/6 mice. Upon MPTP treatment, there is a 6% decrement in nigral DA cell counts in Jackson mice compared to OlaHSD mice. The fact that OlaHSD mice have complete chromosomal absence of α‐synuclein but still succumb to MPTP neurotoxicity suggests a more complex role of α‐synuclein in exacerbating DA cell loss in PD pathology. Lewy body pathology intimates that it is the initial non‐fibrillar α‐synuclein filament type that is the purveyor of cytotoxicity and that the fibrillar aggregates are a residual oligomer that, conversely, may confer neuroprotection (Wakabayashi et al. 2013); an observation that is in conflict with Chandra et al. (2005) noting that mice in which transgenic α‐synuclein is also absent perish within 6 months of age, whereas mice with transgenic α‐synuclein survive for almost 3× as long as their α‐synuclein‐absent counterparts. It is therefore plausible that J6 mice enjoy a higher degree of protection against MPTP neurotoxicity due to their normal α‐synuclein expression levels, whereas the DA neurons in OlaHSD mice might be more vulnerable due to its absence.

The acute toxicity due to MPTP had distinct physical, behavioural and affective effects on each mouse strain, with J6 mice experiencing significant weight loss post‐treatment, whereas OlaHSD mice were unaffected in terms of weight loss. Moreover, OlaHSD mice recovered more rapidly from acute adverse effects post‐MPTP dosing. Basal and reactive gliosis also differed between the sub‐strains. MPTP did not alter the number of reactive astroglia in the SN, but J6 mice had twice as many GFAP‐ir cells compared to OlaHSD mice. In the CPu, MPTP increased the number of GFAP‐ir cells in both sub‐strains, and Iba1‐ir reactivity in the CPu was significantly higher in MPTP‐treated OlaHSD mice than in all other groups. Our study indicates a noticeable rise in microglia in the CPu of OlaHSD mice compared to J6. This increase is further heightened after MPTP treatment, mirroring findings by Siebert et al. (2010), who reported a greater number of macrophages in the sciatic nerve of OlaHSD mice compared to control groups. Previous reports highlight immunological differences between J6 wild‐type and OlaHSD mice that have been fed a high‐fat diet (HFD). Following a HFD, OlaHSD plasma insulin levels increase fourfold and have a higher whole‐body mass and fat mass compared to J6 mice. Liver biopsies from HFD‐fed OlaHSD mice reveal activated transcription factors, including zinc finger protein (GFI‐1) and GATA binding protein 3 (GATA3), both known to influence immune system function, inflammatory processes and stress response (Zeng 2013; Kahle et al. 2013; Wan 2014), suggesting inflammatory abnormalities which may also extend to the central nervous system (Agirman et al. 2021).

### 4.3 Raman Spectra Analysis

**FIGURE 8:**
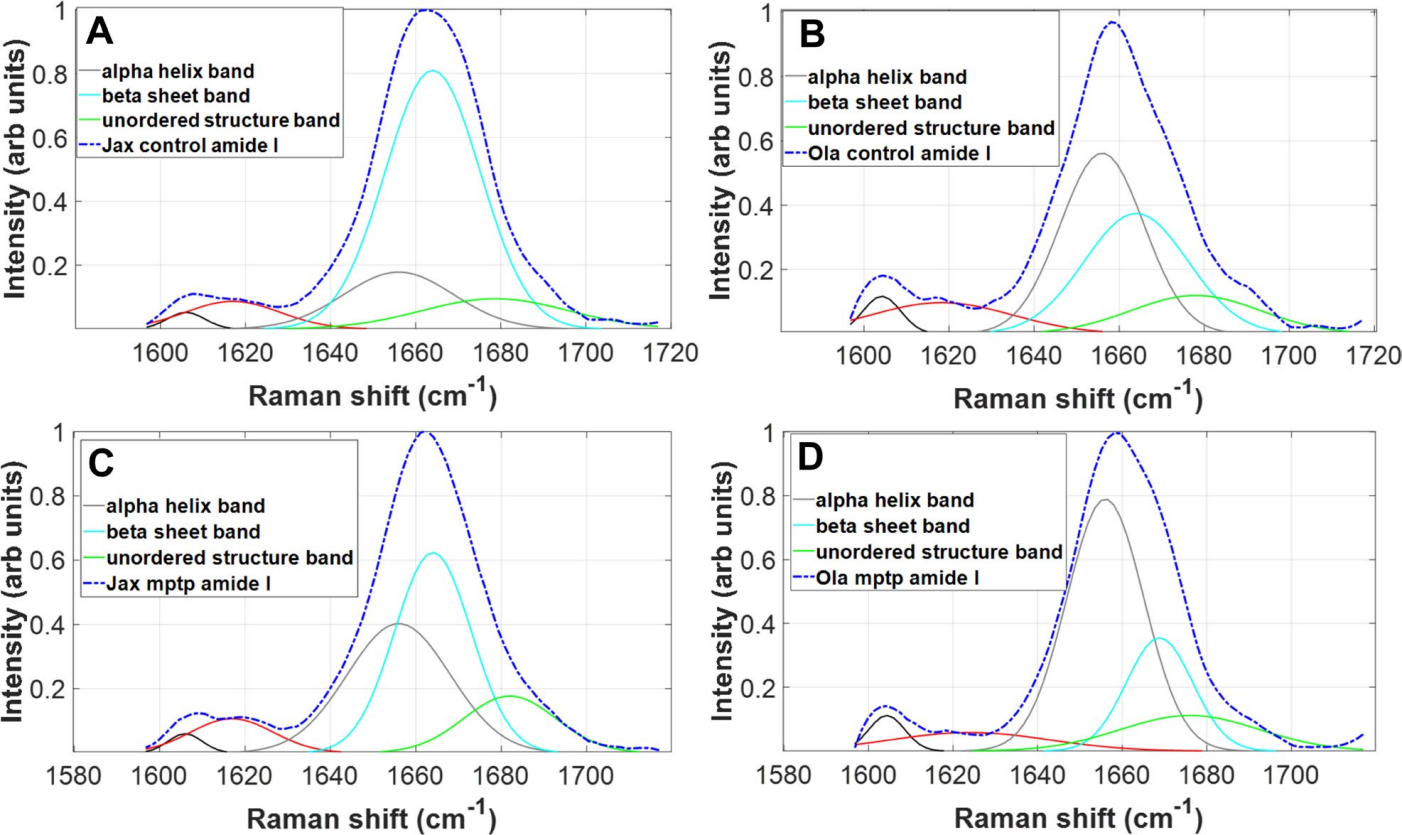
Jackson (J6) versus OlaHSD measurements, Amide‐I peak deconvolutions. Four Gaussian spectra are used for analysis. The panels depict peak analysis of Amide‐I for the average Raman spectrum for control samples (6J, A) and (OlaHSD, B) and MPTP samples (6J, C) and (OlaHSD, D) of the entire scan of each area associated with sub‐strains. The average spectrum displays a reasonable signal‐to‐noise ratio, and Amide‐I peak deconvolution represents a good basis for secondary structure analysis. Constraints in peak deconvolution are introduced relative to peak position according to peak analysis of Maiti et al. (2004).

Raman spectroscopy offers insight into protein's secondary structure in the amide III and amide I spectral regions. The overall contour of the amide I is deconvolved into individual peaks using Gaussian peak fitting total envelope with four peaks (Figure 8) using peak assignment reported in the literature (Maiti et al. 2004; Apetri et al. 2006). The broad peak of amide I houses multiple individual peaks considered for analysis: around 1650 cm^−1^ alpha helix conformation, around 1660 cm^−1^ for beta‐sheet conformation and around 1670 cm^−1^ for polyproline II (Maiti et al. 2004, Apetri et al. 2006). Notable changes were observed in individual peaks after deconvolution analysis that may indicate a different protein conformation. Following deconvolution analysis, we note a distinct ratio in peak heights between the 1650 and 1660 cm^−1^ peaks. Data analysis indicated that proteins in the different sub‐strains contain a different ratio of mixture alpha helix versus beta sheet in their secondary structure. The ratio of different protein conformations between J6 and OlaHSD strain may point to the formation of different protein aggregates in J6, consistent with the onset of Parkinson disease.

A hallmark of Parkinson's disease is the formation of Lewy bodies, which are composed of α‐synuclein protein fibrils. There is no evidence that sub‐acutely MPTP‐treated mice express α‐synuclein protein fibrils. However, MPTP administered for 14 days by slow infusion using osmotic minipumps produced not only dopaminergic neurodegeneration but also the accumulation of α‐synuclein‐ir inclusion bodies (Fornai et al. 2005), suggesting that α‐synuclein protein fibrils might be present at undetectable levels in the sub‐acute experiments carried out here.

It is believed that the spatial conformation of the peptide backbone of the protein adopts a β‐sheet structure (Vidović and Rikalovic 2022). Interestingly, the balance between α‐helix and β‐sheet contributions seems reversed between the J6 and OlaHSD mice sub‐strains. Notably, the peak at approximately 1660 cm^−1^ exhibits the highest intensity in OlaHSD mice, evident in both control and MPTP‐treated groups. The increased footprint signature due to increased levels of β‐sheet structures in OlaHSD mice may indicate a change in conformation of a protein from a monomer to a filament‐like structure. This may be potentially due to the formation of toxic α‐synuclein protein fibrils, which are present in the case of J6 MPTP‐treated, which are absent in OlaHSD mice.

The reverse change in ratio α‐helix/β‐sheet may indicate a transitional phase towards the formation of Lewy bodies. The consistency of these results can be linked to J6 mice being able to normally express α‐synuclein. Another hypothesis is the emergence of a completely new protein structure with a β‐sheet as the new dominant secondary structure in J6 mice. As these results are preliminary, we cannot pinpoint the exact cause. However, we can assert that we observe a consistent shift in protein secondary structure. In addition, the presence of disulphide bond's peaks around 521 cm^−1^ (Rygula et al. 2013) is observed only in the OlaHSD sub‐strain of mice, which may explain the prevalence of stable α‐helix protein conformation.

The comparison between average Raman spectra obtained from the mapped region of SN yields another interesting disparity between sub‐strains—a strong presence of cytochrome *c* in OlaHSD mice highlighted by the two peaks at 747 and 1530 cm^−1^. Cytochrome *c* is involved in α‐synuclein aggregation; however, this is not the case for the Ola sub‐strain because it displays chromosomal deletion of the *Snca1* gene. Other mechanisms could explain what we suspect is an accumulation of cytochrome *c*. There are reports indicating that *Snca1*‐deficient mice sub‐strains may exhibit impairment in the electron transport chain (ETC), which is crucial for the synthesis of ATP (Li et al. 2021). A reduction in ETC activity has been previously linked with an increase in cytochrome *c* release from mitochondria (Oppenheim et al. 2009), which may explain the strong Raman bands recorded in the spectra. Another interesting aspect of cytochrome *c* signal increase was reported by Okada et al. (2011) during cell apoptosis. The redistribution and concentration of the cytochrome *c* content was directed towards the extracellular region. Similarly, the increase in signal that is observed in our data may point to an accumulation of cytochrome *c*.

## 5 Conclusion

The results of this study show that the absence of α‐synuclein has subtle effects on behaviour, susceptibility to MPTP and inflammatory response. These differences may point to a role for α‐synuclein in synaptic transmission, which may eventually lead to compensatory changes that will culminate in the observed changes seen here. However, the absence of a significant difference in susceptibility of the nigrostriatal tract to MPTP between the two mouse strains suggests that at least for neuroprotection studies, they may be used interchangeably. Moreover, the resilience of the OlaHSD strain to MPTP‐induced mortality, which is commonly observed in MPTP mouse models of PD, suggests that this strain may offer distinct advantages as far as the principle of 3Rs is concerned over the commonly used 6J strains, especially with regard to neuroprotection studies in this species.

Raman spectroscopy revealed a change in protein conformation from alpha helix to beta sheet in the case of the Jax sub‐strain, indicative of the formation of an intermediary amyloid structure. Collectively, the observed subtle behavioural and biochemical alterations appear to correlate with the corresponding variations in Raman spectral signatures acquired during the measurements. We can therefore conclude that Raman spectroscopy can be utilised to detect changes not only in the absence of α‐synuclein but also in α‐synuclein overexpression in brain and other Parkinsonian tissues. As such, Raman spectroscopy could provide a novel tool for diagnosis of PD from non‐brain tissues.

## Author Contributions

**W. H. Powell:** investigation, formal analysis, data curation, writing – original draft, methodology. **A. Ghita:** conceptualization, visualization, validation, writing – review and editing, formal analysis, resources. **F. C. Pascut:** methodology, software, validation, investigation. **K. F. Webb:** conceptualization, visualization, writing – review and editing, project administration, supervision, methodology, validation. **A. Newman‐Tancredi:** conceptualization, writing – review and editing, visualization, project administration, supervision. **M. M. Iravani:** conceptualization, investigation, funding acquisition, writing – review and editing, resources, supervision, data curation, methodology, validation, formal analysis, project administration.

## Conflicts of Interest

The authors declare no conflicts of interest.

## Peer Review

The peer review history for this article is available at https://www.webofscience.com/api/gateway/wos/peer‐review/10.1111/jnc.70201.

## Data Availability

The data that support the findings of this study are available from the corresponding author upon reasonable request.
